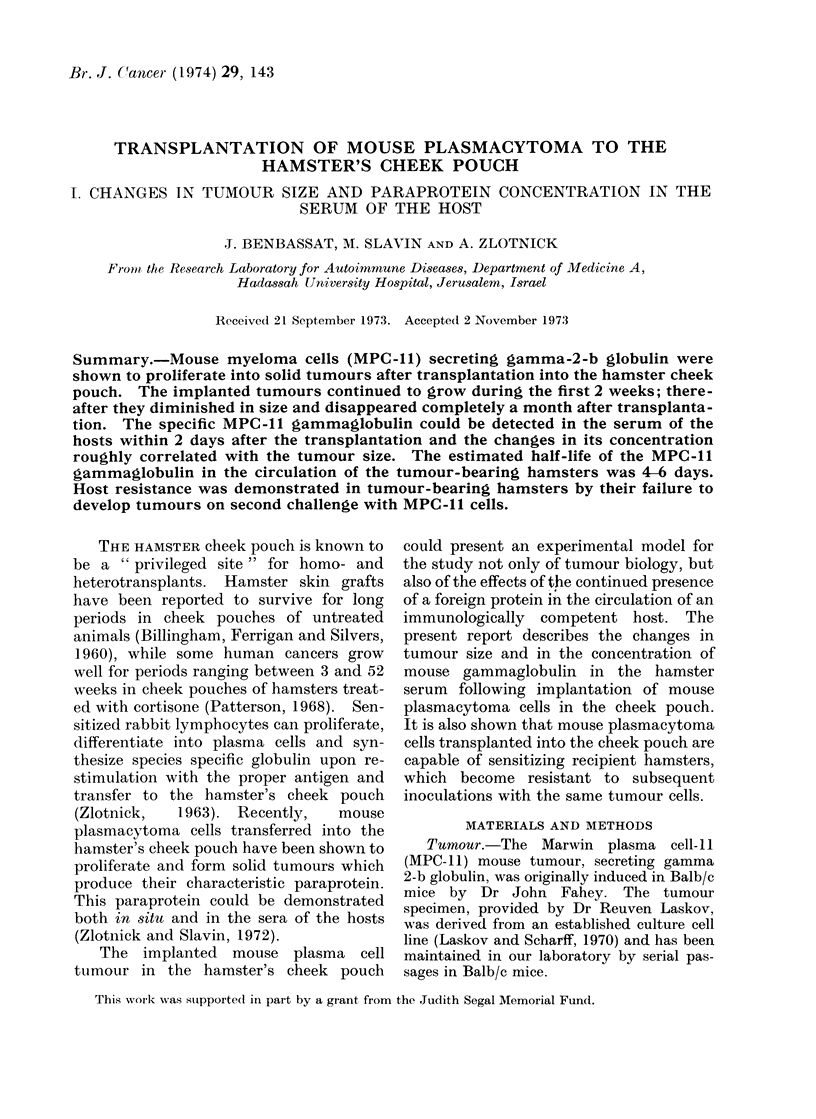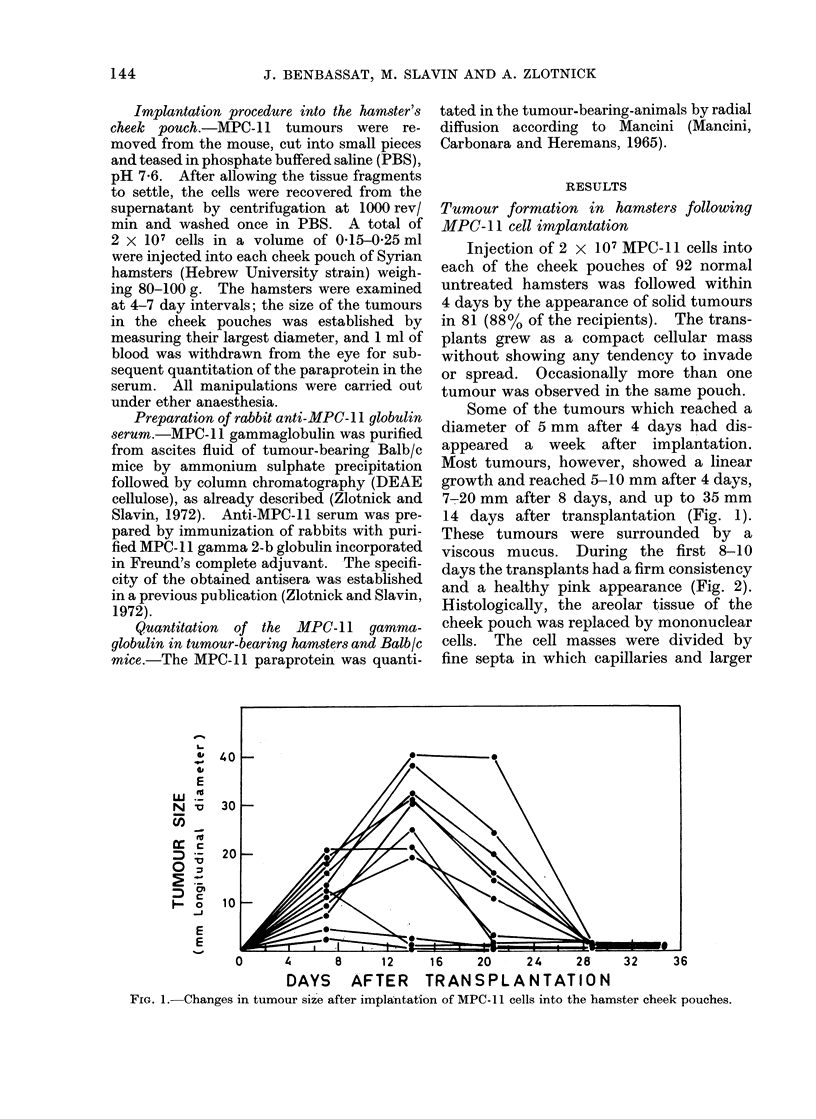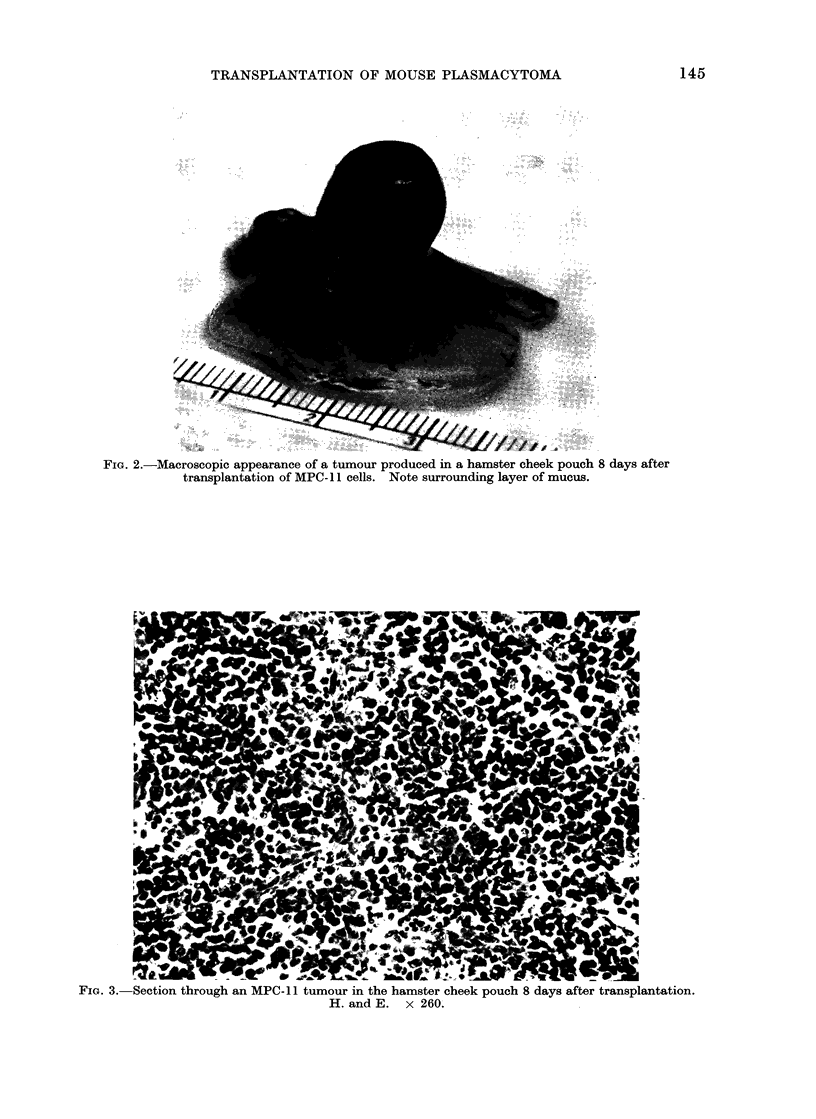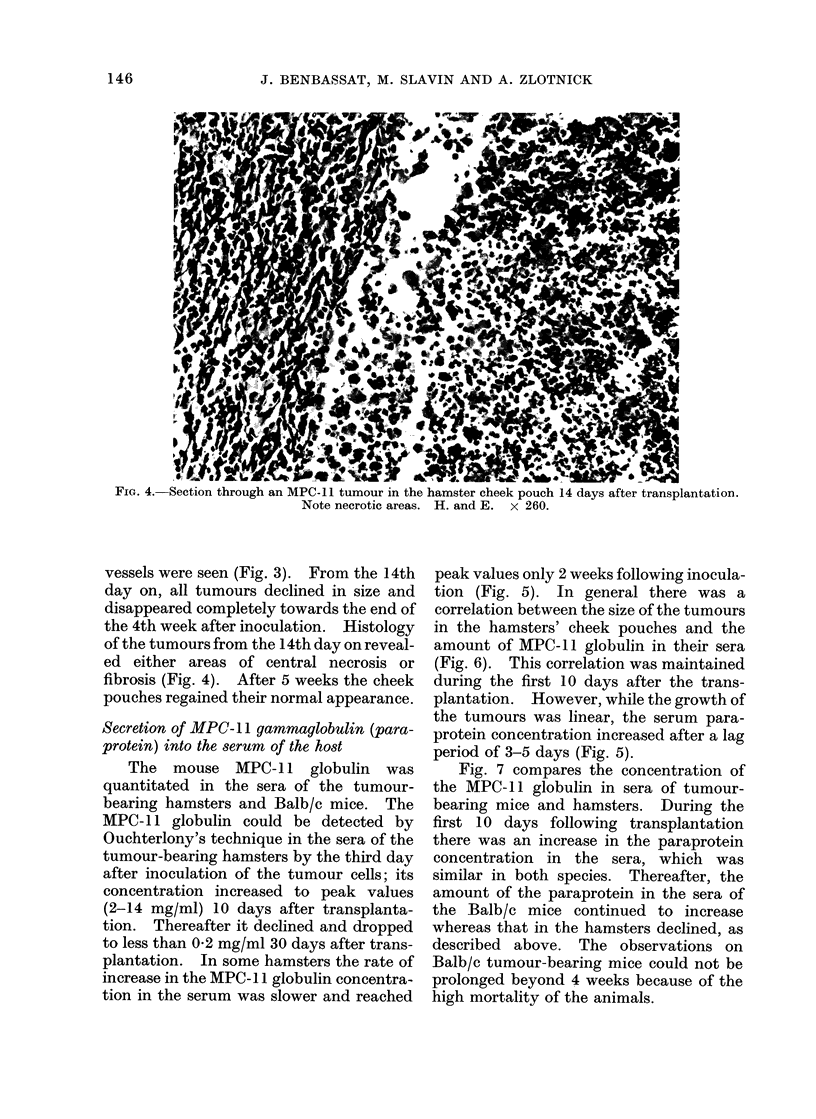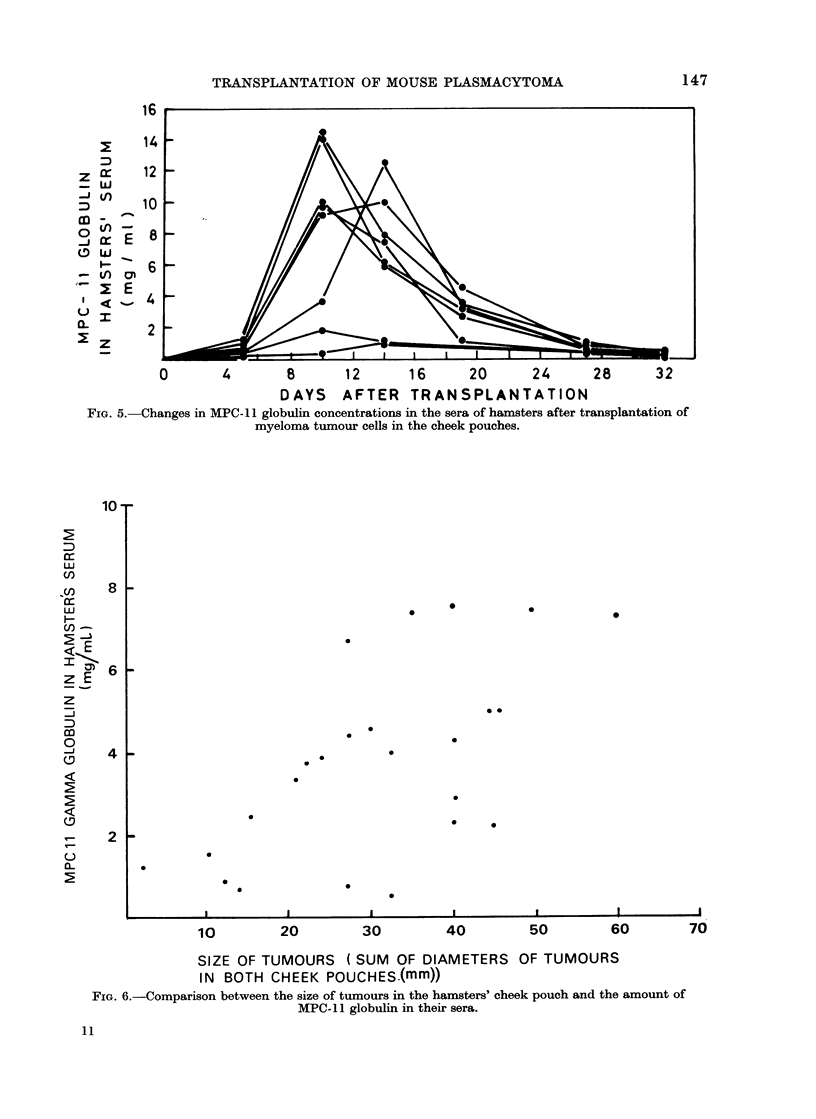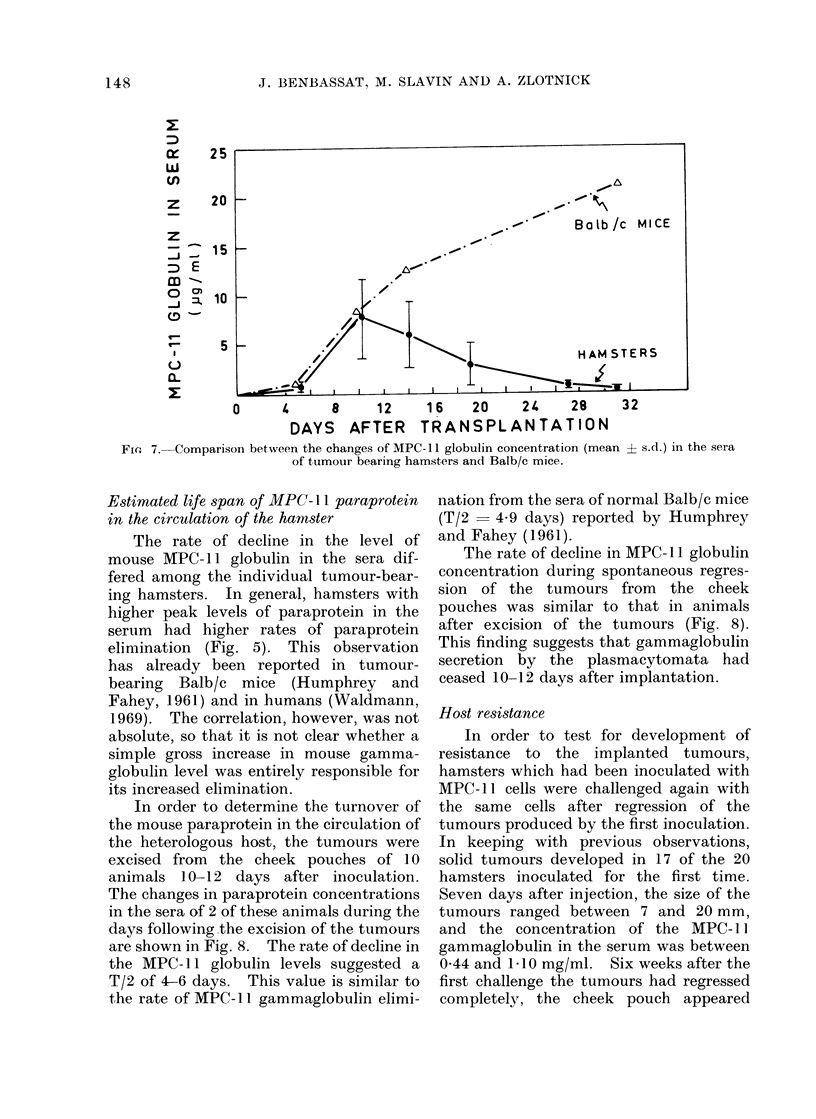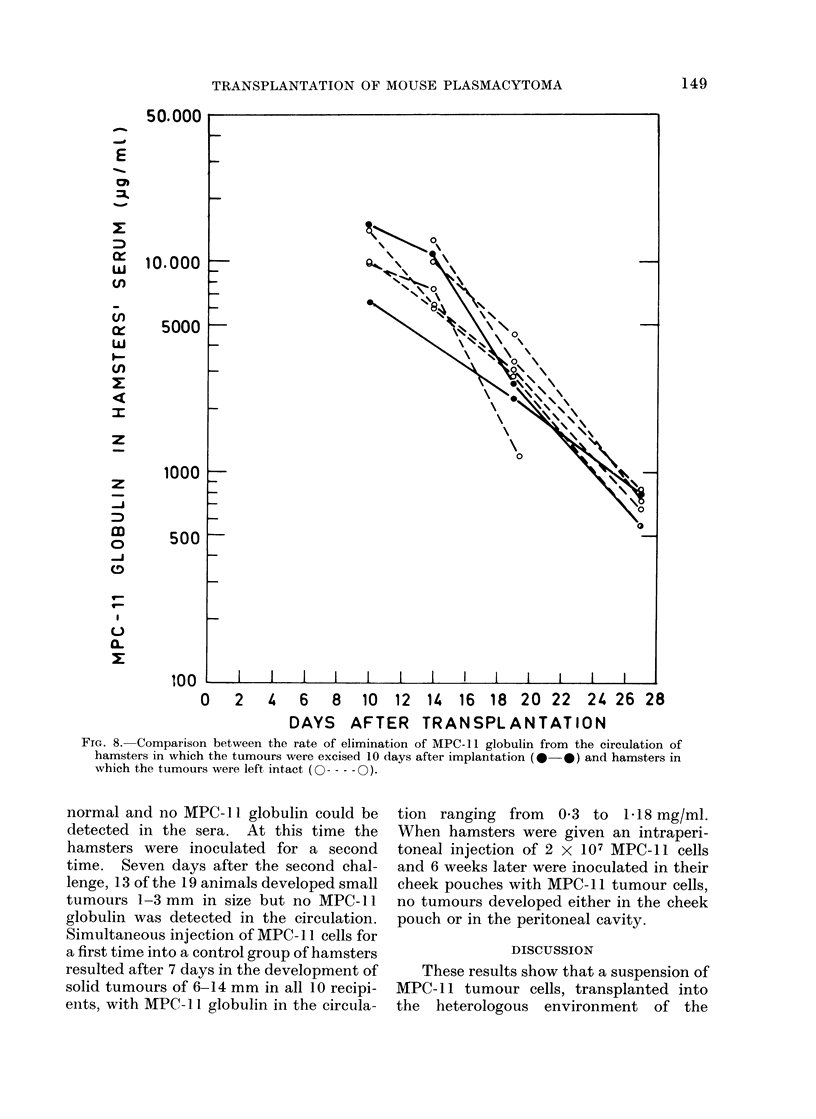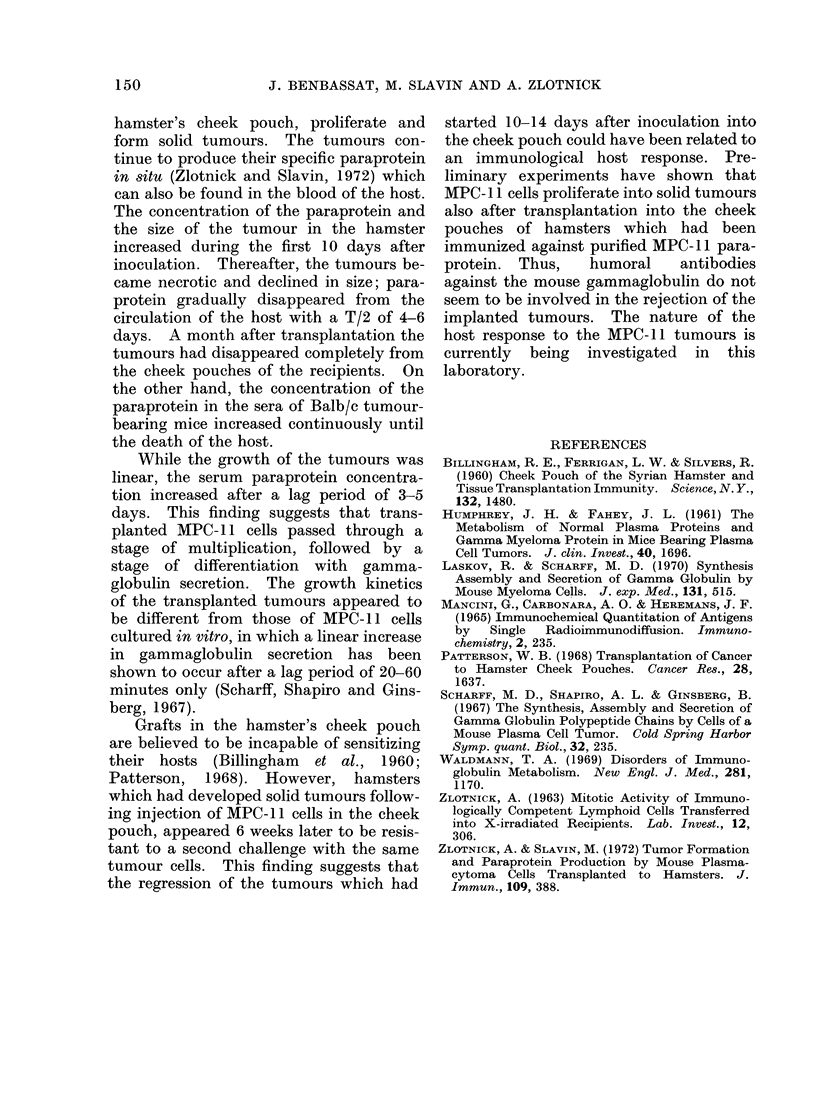# Transplantation of Mouse Plasmacytoma to the Hamster's Cheek Pouch

**DOI:** 10.1038/bjc.1974.50

**Published:** 1974-02

**Authors:** J. Benbassat, M. Slavin, A. Zlotnick

## Abstract

**Images:**


*Br. J. Cancer* (1974) **29**, 143

### TRANSPLANTATION OF MOUSE PLASMACYTOMA TO THE HAMSTER'S CHEEK POUCH

### I. CHANGES IN TUMOUR SIZE AND PARAPROTEIN CONCENTRATION IN THE SERUM OF THE HOST

J. BENBASSAT, M. SLAVIN AND A. ZLOTNICK

*From the Research Laboratory for Autoimmune Diseases, Department of Medicine A, Hadassah University Hospital, Jerusalem, Israel*

Received 21 September 1973. Accepted 2 November 1973

**Summary.—Mouse myeloma cells (MPC-11) secreting gamma-2-b globulin were shown to proliferate into solid tumours after transplantation into the hamster cheek pouch. The implanted tumours continued to grow during the first 2 weeks; thereafter they diminished in size and disappeared completely a month after transplantation. The specific MPC-11 gammaglobulin could be detected in the serum of the hosts within 2 days after the transplantation and the changes in its concentration roughly correlated with the tumour size. The estimated half-life of the MPC-11 gammaglobulin in the circulation of the tumour-bearing hamsters was 4–6 days. Host resistance was demonstrated in tumour-bearing hamsters by their failure to develop tumours on second challenge with MPC-11 cells.**

THE HAMSTER cheek pouch is known to be a "privileged site" for homo- and heterotransplants. Hamster skin grafts have been reported to survive for long periods in cheek pouches of untreated animals (Billingham, Ferrigan and Silvers, 1960), while some human cancers grow well for periods ranging between 3 and 52 weeks in cheek pouches of hamsters treated with cortisone (Patterson, 1968). Sensitized rabbit lymphocytes can proliferate, differentiate into plasma cells and synthesize species specific globulin upon restimulation with the proper antigen and transfer to the hamster's cheek pouch (Zlotnick, 1963). Recently, mouse plasmacytoma cells transferred into the hamster's cheek pouch have been shown to proliferate and form solid tumours which produce their characteristic paraprotein. This paraprotein could be demonstrated both *in situ* and in the sera of the hosts (Zlotnick and Slavin, 1972).

The implanted mouse plasma cell tumour in the hamster's cheek pouch could present an experimental model for the study not only of tumour biology, but also of the effects of the continued presence of a foreign protein in the circulation of an immunologically competent host. The present report describes the changes in tumour size and in the concentration of mouse gammaglobulin in the hamster serum following implantation of mouse plasmacytoma cells in the cheek pouch. It is also shown that mouse plasmacytoma cells transplanted into the cheek pouch are capable of sensitizing recipient hamsters, which become resistant to subsequent inoculations with the same tumour cells.

### MATERIALS AND METHODS

*Tumour.*—The Marwin plasma cell-11 (MPC-11) mouse tumour, secreting gamma 2-b globulin, was originally induced in Balb/c mice by Dr John Fahey. The tumour specimen, provided by Dr Reuven Laskov, was derived from an established culture cell line (Laskov and Scharff, 1970) and has been maintained in our laboratory by serial passages in Balb/c mice.

This work was supported in part by a grant from the Judith Segal Memorial Fund.

*Implantation procedure into the hamster's cheek pouch.*—MPC-11 tumours were removed from the mouse, cut into small pieces and teased in phosphate buffered saline (PBS), pH 7·6. After allowing the tissue fragments to settle, the cells were recovered from the supernatant by centrifugation at 1000 rev/min and washed once in PBS. A total of $2 \times 10^7$ cells in a volume of 0·15–0·25 ml were injected into each cheek pouch of Syrian hamsters (Hebrew University strain) weighing 80–100 g. The hamsters were examined at 4–7 day intervals; the size of the tumours in the cheek pouches was established by measuring their largest diameter, and 1 ml of blood was withdrawn from the eye for subsequent quantitation of the paraprotein in the serum. All manipulations were carried out under ether anaesthesia.

*Preparation of rabbit anti-MPC-11 globulin serum.*—MPC-11 gammaglobulin was purified from ascites fluid of tumour-bearing Balb/c mice by ammonium sulphate precipitation followed by column chromatography (DEAE cellulose), as already described (Zlotnick and Slavin, 1972). Anti-MPC-11 serum was prepared by immunization of rabbits with purified MPC-11 gamma 2-b globulin incorporated in Freund's complete adjuvant. The specificity of the obtained antisera was established in a previous publication (Zlotnick and Slavin, 1972).

*Quantitation of the MPC-11 gammaglobulin in tumour-bearing hamsters and Balb/c mice.*—The MPC-11 paraprotein was quantitated in the tumour-bearing-animals by radial diffusion according to Mancini (Mancini, Carbonara and Heremans, 1965).

### RESULTS

*Tumour formation in hamsters following MPC-11 cell implantation*

Injection of $2 \times 10^7$ MPC-11 cells into each of the cheek pouches of 92 normal untreated hamsters was followed within 4 days by the appearance of solid tumours in 81 (88% of the recipients). The transplants grew as a compact cellular mass without showing any tendency to invade or spread. Occasionally more than one tumour was observed in the same pouch.

Some of the tumours which reached a diameter of 5 mm after 4 days had disappeared a week after implantation. Most tumours, however, showed a linear growth and reached 5–10 mm after 4 days, 7–20 mm after 8 days, and up to 35 mm 14 days after transplantation (Fig. 1). These tumours were surrounded by a viscous mucus. During the first 8–10 days the transplants had a firm consistency and a healthy pink appearance (Fig. 2). Histologically, the areolar tissue of the cheek pouch was replaced by mononuclear cells. The cell masses were divided by fine septa in which capillaries and larger

Fig. 1.—Changes in tumour size after implantation of MPC-11 cells into the hamster cheek pouches.

FIG. 2.—Macroscopic appearance of a tumour produced in a hamster cheek pouch 8 days after transplantation of MPC-11 cells. Note surrounding layer of mucus.

FIG. 3.—Section through an MPC-11 tumour in the hamster cheek pouch 8 days after transplantation. H. and E. × 260.

FIG. 4.—Section through an MPC-11 tumour in the hamster cheek pouch 14 days after transplantation. Note necrotic areas. H. and E. × 260.

vessels were seen (Fig. 3). From the 14th day on, all tumours declined in size and disappeared completely towards the end of the 4th week after inoculation. Histology of the tumours from the 14th day on revealed either areas of central necrosis or fibrosis (Fig. 4). After 5 weeks the cheek pouches regained their normal appearance.

*Secretion of MPC-*11 *gammaglobulin* (*paraprotein*) *into the serum of the host*

The mouse MPC-11 globulin was quantitated in the sera of the tumour-bearing hamsters and Balb/c mice. The MPC-11 globulin could be detected by Ouchterlony's technique in the sera of the tumour-bearing hamsters by the third day after inoculation of the tumour cells; its concentration increased to peak values (2–14 mg/ml) 10 days after transplantation. Thereafter it declined and dropped to less than 0·2 mg/ml 30 days after transplantation. In some hamsters the rate of increase in the MPC-11 globulin concentration in the serum was slower and reached peak values only 2 weeks following inoculation (Fig. 5). In general there was a correlation between the size of the tumours in the hamsters' cheek pouches and the amount of MPC-11 globulin in their sera (Fig. 6). This correlation was maintained during the first 10 days after the transplantation. However, while the growth of the tumours was linear, the serum paraprotein concentration increased after a lag period of 3–5 days (Fig. 5).

Fig. 7 compares the concentration of the MPC-11 globulin in sera of tumour-bearing mice and hamsters. During the first 10 days following transplantation there was an increase in the paraprotein concentration in the sera, which was similar in both species. Thereafter, the amount of the paraprotein in the sera of the Balb/c mice continued to increase whereas that in the hamsters declined, as described above. The observations on Balb/c tumour-bearing mice could not be prolonged beyond 4 weeks because of the high mortality of the animals.

FIG. 5.—Changes in MPC-11 globulin concentrations in the sera of hamsters after transplantation of myeloma tumour cells in the cheek pouches.

FIG. 6.—Comparison between the size of tumours in the hamsters' cheek pouch and the amount of MPC-11 globulin in their sera.

FIG 7.—Comparison between the changes of MPC-11 globulin concentration (mean ± s.d.) in the sera of tumour bearing hamsters and Balb/c mice.

*Estimated life span of MPC-*11 *paraprotein in the circulation of the hamster*

The rate of decline in the level of mouse MPC-11 globulin in the sera differed among the individual tumour-bearing hamsters. In general, hamsters with higher peak levels of paraprotein in the serum had higher rates of paraprotein elimination (Fig. 5). This observation has already been reported in tumour-bearing Balb/c mice (Humphrey and Fahey, 1961) and in humans (Waldmann, 1969). The correlation, however, was not absolute, so that it is not clear whether a simple gross increase in mouse gammaglobulin level was entirely responsible for its increased elimination.

In order to determine the turnover of the mouse paraprotein in the circulation of the heterologous host, the tumours were excised from the cheek pouches of 10 animals 10–12 days after inoculation. The changes in paraprotein concentrations in the sera of 2 of these animals during the days following the excision of the tumours are shown in Fig. 8. The rate of decline in the MPC-11 globulin levels suggested a T/2 of 4–6 days. This value is similar to the rate of MPC-11 gammaglobulin elimination from the sera of normal Balb/c mice (T/2 = 4·9 days) reported by Humphrey and Fahey (1961).

The rate of decline in MPC-11 globulin concentration during spontaneous regression of the tumours from the cheek pouches was similar to that in animals after excision of the tumours (Fig. 8). This finding suggests that gammaglobulin secretion by the plasmacytomata had ceased 10–12 days after implantation.

*Host resistance*

In order to test for development of resistance to the implanted tumours, hamsters which had been inoculated with MPC-11 cells were challenged again with the same cells after regression of the tumours produced by the first inoculation. In keeping with previous observations, solid tumours developed in 17 of the 20 hamsters inoculated for the first time. Seven days after injection, the size of the tumours ranged between 7 and 20 mm, and the concentration of the MPC-11 gammaglobulin in the serum was between 0·44 and 1·10 mg/ml. Six weeks after the first challenge the tumours had regressed completely, the cheek pouch appeared

FIG. 8.—Comparison between the rate of elimination of MPC-11 globulin from the circulation of hamsters in which the tumours were excised 10 days after implantation (●—●) and hamsters in which the tumours were left intact (○- - - -○).

normal and no MPC-11 globulin could be detected in the sera. At this time the hamsters were inoculated for a second time. Seven days after the second challenge, 13 of the 19 animals developed small tumours 1–3 mm in size but no MPC-11 globulin was detected in the circulation. Simultaneous injection of MPC-11 cells for a first time into a control group of hamsters resulted after 7 days in the development of solid tumours of 6–14 mm in all 10 recipients, with MPC-11 globulin in the circulation ranging from 0·3 to 1·18 mg/ml. When hamsters were given an intraperitoneal injection of $2 \times 10^7$ MPC-11 cells and 6 weeks later were inoculated in their cheek pouches with MPC-11 tumour cells, no tumours developed either in the cheek pouch or in the peritoneal cavity.

### DISCUSSION

These results show that a suspension of MPC-11 tumour cells, transplanted into the heterologous environment of the hamster's cheek pouch, proliferate and form solid tumours. The tumours continue to produce their specific paraprotein *in situ* (Zlotnick and Slavin, 1972) which can also be found in the blood of the host. The concentration of the paraprotein and the size of the tumour in the hamster increased during the first 10 days after inoculation. Thereafter, the tumours became necrotic and declined in size; paraprotein gradually disappeared from the circulation of the host with a T/2 of 4–6 days. A month after transplantation the tumours had disappeared completely from the cheek pouches of the recipients. On the other hand, the concentration of the paraprotein in the sera of Balb/c tumour-bearing mice increased continuously until the death of the host.

While the growth of the tumours was linear, the serum paraprotein concentration increased after a lag period of 3–5 days. This finding suggests that transplanted MPC-11 cells passed through a stage of multiplication, followed by a stage of differentiation with gammaglobulin secretion. The growth kinetics of the transplanted tumours appeared to be different from those of MPC-11 cells cultured *in vitro*, in which a linear increase in gammaglobulin secretion has been shown to occur after a lag period of 20–60 minutes only (Scharff, Shapiro and Ginsberg, 1967).

Grafts in the hamster's cheek pouch are believed to be incapable of sensitizing their hosts (Billingham *et al.*, 1960; Patterson, 1968). However, hamsters which had developed solid tumours following injection of MPC-11 cells in the cheek pouch, appeared 6 weeks later to be resistant to a second challenge with the same tumour cells. This finding suggests that the regression of the tumours which had started 10–14 days after inoculation into the cheek pouch could have been related to an immunological host response. Preliminary experiments have shown that MPC-11 cells proliferate into solid tumours also after transplantation into the cheek pouches of hamsters which had been immunized against purified MPC-11 paraprotein. Thus, humoral antibodies against the mouse gammaglobulin do not seem to be involved in the rejection of the implanted tumours. The nature of the host response to the MPC-11 tumours is currently being investigated in this laboratory.

### REFERENCES

BILLINGHAM, R. E., FERRIGAN, L. W. & SILVERS, R. (1960) Cheek Pouch of the Syrian Hamster and Tissue Transplantation Immunity. *Science, N.Y.*, **132**, 1480.

HUMPHREY, J. H. & FAHEY, J. L. (1961) The Metabolism of Normal Plasma Proteins and Gamma Myeloma Protein in Mice Bearing Plasma Cell Tumors. *J. clin. Invest.*, **40**, 1696.

LASKOV, R. & SCHARFF, M. D. (1970) Synthesis Assembly and Secretion of Gamma Globulin by Mouse Myeloma Cells. *J. exp. Med.*, **131**, 515.

MANCINI, G., CARBONARA, A. O. & HEREMANS, J. F. (1965) Immunochemical Quantitation of Antigens by Single Radioimmunodiffusion. *Immunochemistry*, **2**, 235.

PATTERSON, W. B. (1968) Transplantation of Cancer to Hamster Cheek Pouches. *Cancer Res.*, **28**, 1637.

SCHARFF, M. D., SHAPIRO, A. L. & GINSBERG, B. (1967) The Synthesis, Assembly and Secretion of Gamma Globulin Polypeptide Chains by Cells of a Mouse Plasma Cell Tumor. *Cold Spring Harbor Symp. quant. Biol.*, **32**, 235.

WALDMANN, T. A. (1969) Disorders of Immunoglobulin Metabolism. *New Engl. J. Med.*, **281**, 1170.

ZLOTNICK, A. (1963) Mitotic Activity of Immunologically Competent Lymphoid Cells Transferred into X-irradiated Recipients. *Lab. Invest.*, **12**, 306.

ZLOTNICK, A. & SLAVIN, M. (1972) Tumor Formation and Paraprotein Production by Mouse Plasmacytoma Cells Transplanted to Hamsters. *J. Immun.*, **109**, 388.